# Low-cost double pendulum for high-quality data collection with open-source video tracking and analysis

**DOI:** 10.1016/j.ohx.2020.e00138

**Published:** 2020-09-11

**Authors:** Audun D. Myers, Joshua R. Tempelman, David Petrushenko, Firas A. Khasawneh

**Affiliations:** Department of Mechanical Engineering at Michigan State University, Department of Mechanical Engineering at Virginia Tech, United States

**Keywords:** Double pendulum, Pendulum, Tracking, Occlusions

## Abstract

The double pendulum is a system that manifests fascinating non-linear behavior. This made it a popular tool in academic settings for illustrating the intricate response of a seemingly simple physical apparatus, or to validate tools for studying nonlinear phenomena. In addition, the double pendulum is also widely used in several modeling applications including robotics and human locomotion analysis. However, surprisingly, there is a lack of a thoroughly documented hardware that enables designing, building, and reliably tracking and collecting data from a double pendulum.

This paper provides comprehensive documentation of a research quality bench top double pendulum. The contributions of our work include (1) providing detailed CAD drawings, part lists, and assembly instructions for building a low friction double pendulum. (2) A new tracking algorithm written in Python for tracking the position of both links of the double pendulum. This algorithm measures the angles of the links by examining each frame, and computes uncertainties in the measured angles by following several trackers on each link. Additionally, our tracking algorithm bypasses the data transmission difficulties caused by instrumenting the bottom link with physical sensors. (3) A derivation of the equations of motion of the actual physical system. (4) A description of the process (with provided Python code) for extracting the model parameters—e.g., damping—with error bounds from physical measurements.

## Graphical abstract

**Figure:**
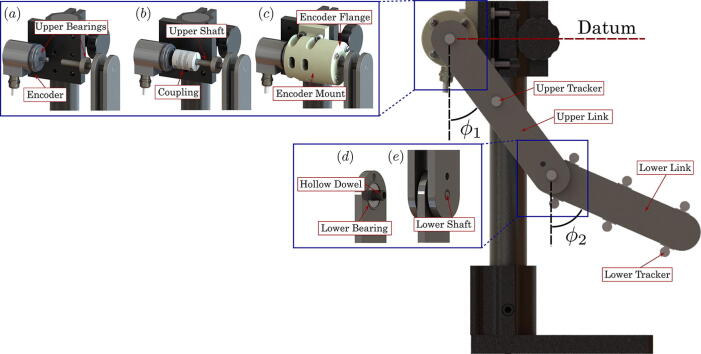

.

**Table:** 

**Hardware name**	*Research Quality Double Pendulum*
**Subject area**	• *Mechanical Engineering* • *Nonlinear Vibrations* • *Computer Vision*
**Hardware type**	• *Double Pendulum* • *Benchtop Experiment*
**Open source license**	GNU General Public License Version 3
**Cost of hardware**	$591
**Source file repository**	https://doi.org/10.17632/z4hvxjgtbz.2

## 1 Hardware in context

The double pendulum is widely used in education, research, and applications. For example, the double pendulum is a staple benchtop experiment for introducing and studying chaos and state transitions. It has also been used to study chaos both experimentally [1], [2], [3] and numerically [4], [5]. The double pendulum was successfully used as a modeling tool in robotics and bio-mechanics applications. Specifically, a double pendulum model has been used to analyze the control aspects of a driven cart robot [6], flexible arm robotics [7], and shipboard cranes [8]. The double pendulum has also been used as a simple experiment for chaos [9], [10] and position [11], [12] control applications. Outside of robotics, the double pendulum has been leveraged to model animal and human dynamics via an analysis of the locomotion of the human leg swing [13], gait dynamics [14], and even the golf swing [15]. The double pendulum has also been used to test model discovery algorithms due to its complicated and rich dynamics [16], [17], [18].

Despite the popularity of the double pendulum, a thorough process for double pendulum manufacturing and the subsequent data collection and analysis is still lacking. For example, in the double pendulum experiment repository by Andrzej Morawa and Karol Strama [19], an experimental design is proposed for tracking each link via video data for a wooden double pendulum. However, both the design and the tracking algorithm are limited to motion without occlusions such that the lower link does not complete any full rotations or go behind the upper link. This significantly limits the applications for their experimental design. Additionally, a manufacturing process is not provided, which also makes replicating their experiment difficult. For another example, Schmidt et al. [16] developed a double pendulum tracked with video data which could have a full range of motion, but they had to omit frames where the trackers were occluded. This limited the amount of information available from an experiment. As a further limitation, many designs found commercially or in the literature do not try to maximize the amount of time the system is in motion. This results in very short time series consisting of pieces with different dynamic behavior that cannot be used to study the underlying rich dynamics.

In this work we address this issue by providing a comprehensive, open-source design document for manufacturing, operating, and collecting high-quality double pendulum data. The main tool for data collection relies on instrumenting the pendulum with reflective markers and capturing their motion with a high-speed camera. However, we include in our design an optional encoder for the top link that we used for validating the tracking algorithms.

## 2 Hardware description

**Fig. 1:**
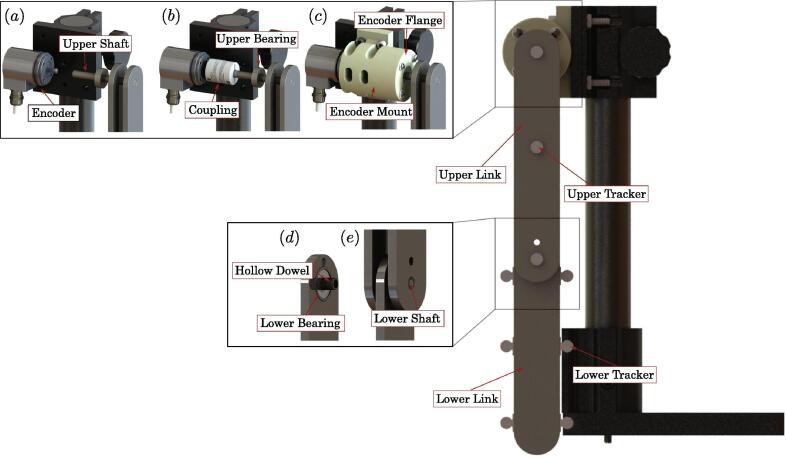
CAD rendering of bench top double pendulum with emphasized key components.

The double pendulum (see Fig. 1) we present in this paper is a research quality benchtop experiment for studying dynamical systems and chaotic behavior. The design used provides low friction through minimizing the number of needed bearings, and utilizing low-friction skateboard bearings.

This experimental design has two methods for tracking: (1) an (optional) encoder coupled to the upper link for measuring the angular displacement, and (2) video tracking of reflective markers for measuring the angular displacement of both the upper and lower links. The encoder data allowed us to validate the tracking algorithm, but it is not required. We stress that the tracking algorithm, which utilizes Open Source Computer Vision Library (OpenCV) deals with difficult targets due to the double pendulum nonlinearity, and the occlusions caused by some of the markers on the bottom link passing behind the top link.

The following is a summary list of key descriptions of the double pendulum:

• Research quality double pendulum for studying dynamical systems and chaos with a low friction coefficient.
• The simple design allows for easy reproduction, and enables convenient, accurate, and reproducible calibration curves when using the optional encoder. The details of a calibration apparatus for reproducible results, which was used for a single pendulum apparatus [20], can be found in [21].
• The reflective tracker design allows the reliable tracking of both pendulum arms, which can be validated using the built-in encoder. The tracking algorithm also automatically computes the uncertainty in measuring the swing angles through tracking multiple lines on each link.
• The described modeling process allows coupling the hardware with its governing equations of motion.

## 3 Design files

**Fig. 2:**
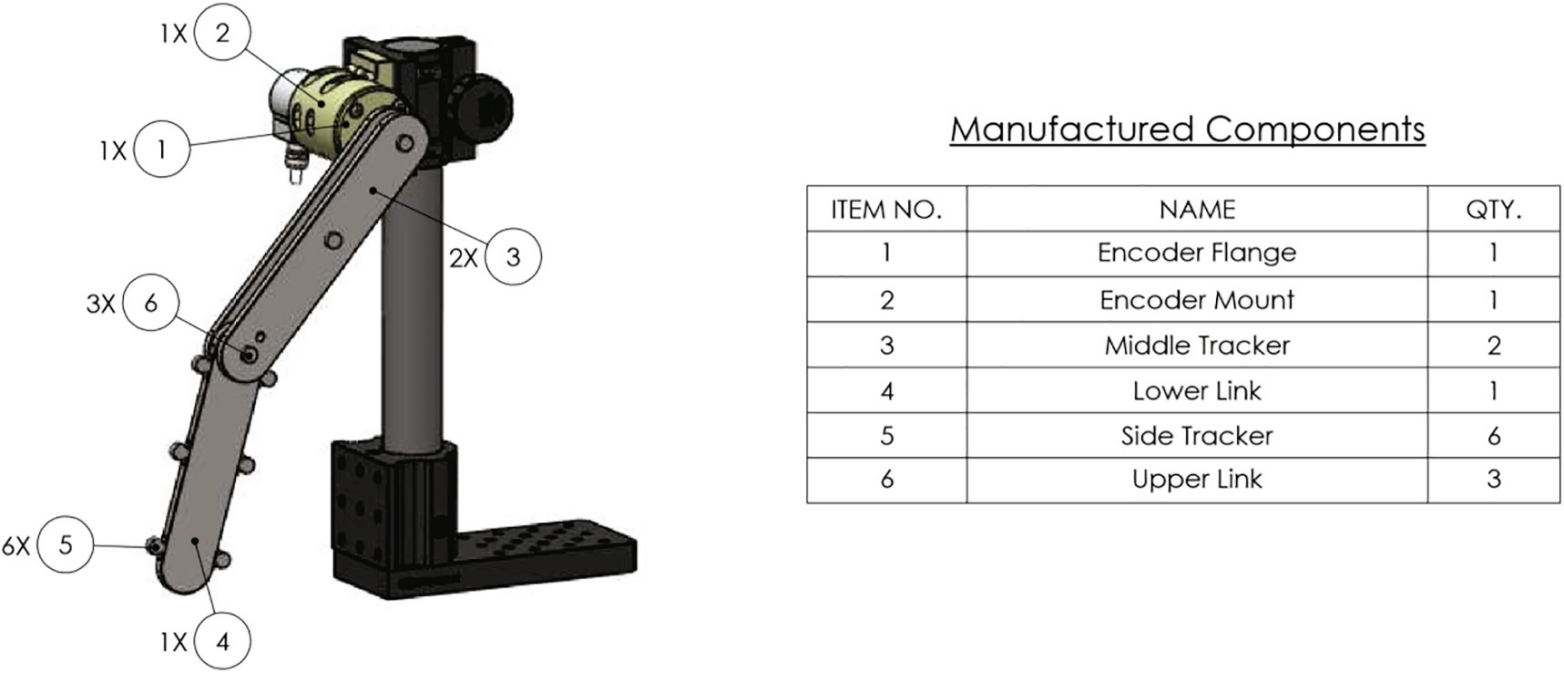
Mechanical drawing overview figure for manufactured components.

This section provides descriptions and links to the design files used for both manufacturing and data acquisition. To begin we will first introduce all the manufactured components (see Fig. 2), which will be followed then by a summary of the software used for data acquisition from the encoder and the novel tracking algorithm for non-linear rigid-body dynamics with occlusions.

### 3.1 Machined components

**Fig. 3:**
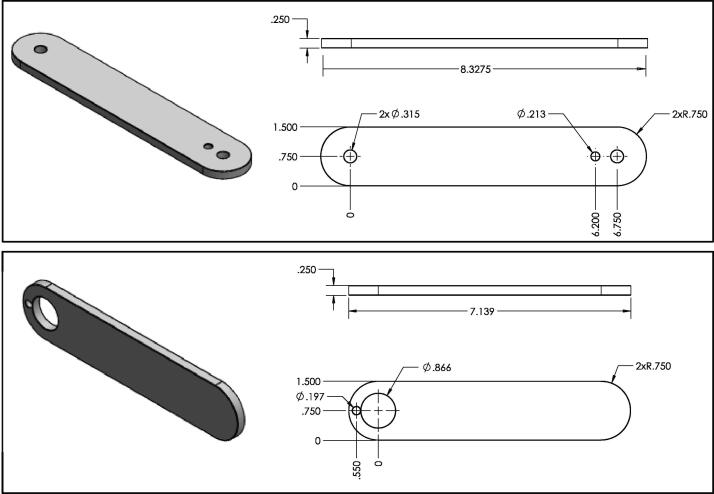
Mechanical drawing of the machined upper (top figure) and lower (bottom figure) links (items 4 and 6 from Fig. 2). All dimensions are in inches.

The two components which require machining to produce are the upper and lower links of the pendulum (see Fig. 3). Both of these components are made from aluminum 6061, which were manufactured using a CNC machine. However, these two components could be manufactured from another material or possibly 3D printed if desired. Either way, similar dynamics can still be obtained by matching the mass moment of inertia to the current linkage. More details on the manufacturing of the pendulum links are described in Section 5.

### 3.2 3D printed components

**Fig. 4:**
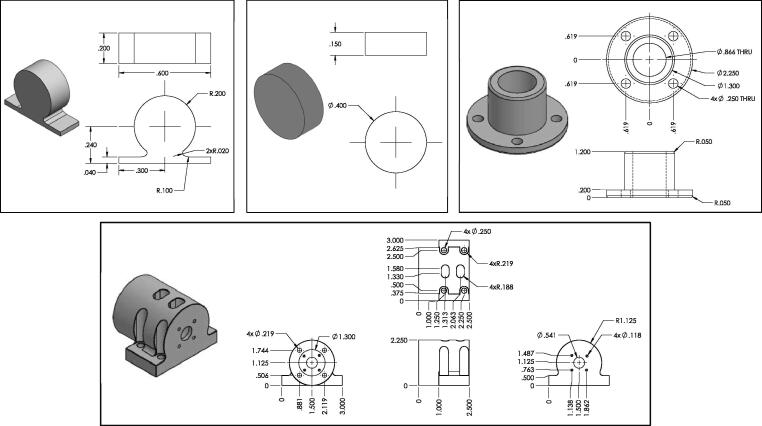
Mechanical drawing of 3D printed components (dimensions are in inches): (top left and center) 3D printed upper and lower pendulum arm trackers (items 3 and 5 from Fig. 2), top right) encoder mount flange (item 1 from Fig. 2bottom) encoder mount housing (item 2 from Fig. 2).

There are a total of four 3D printed components used in the assembly. The first two are the 3D printed tracker markers (see top left and center of Fig. 4). These are 3D printed out of Polylactic Acid (PLA) with 20 percent infill to reduce their mass. The other two components are used to form the encoder and upper bearing housing, which are printed from PLA at 100 percent infill to increase the component stiffness (see top right and bottom of Fig. 4). All of these 3D printed components have their respective dimensions (inches) provided in Fig. 4.

### 3.3 Software

**Fig. 5:**
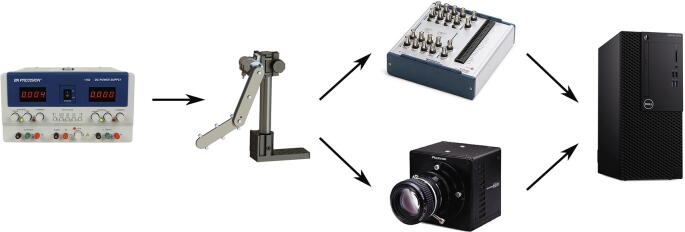
Flow chart of electronics used: 24 V DC from power supply to power pendulum encoder, which is recorded using a DAQ and with a high speed camera. The recorded data is then stored directly on a PC.

Before going into detail of the software used, it is helpful to provide a simple flow chart of the electronics and data collected systems used in a sample experiment. Fig. 5 shows a 24 V DC power supply to power the pendulum encoder, which is recorded using a DAQ and a high speed camera. The recorded data is then stored directly on a PC. We now introduce the software used to process the data from both the encoder and the video data.

#### 3.3.1 Encoder data acquisition software

The optional encoder data was collected from the data acquisition system (NI USB-6356) using a built in MATLAB® NI-DAQmx Toolbox. Example MatLab code implementing the toolbox for recording data from the encoder is provided with a convenient graphics-user-interface. However, the data acquisition from the encoder could be achieved using other software or data acquisition cards—We are only reporting one possible way, and the method that we used. However, for similar accuracy we suggest using an encoder with a Counts Per Revolution (CPR) value ⩾1000. A discussion of the data collection and calibration of the encoder is also provided in Appendix B.

#### 3.3.2 Tracking algorithm

**Fig. 6:**
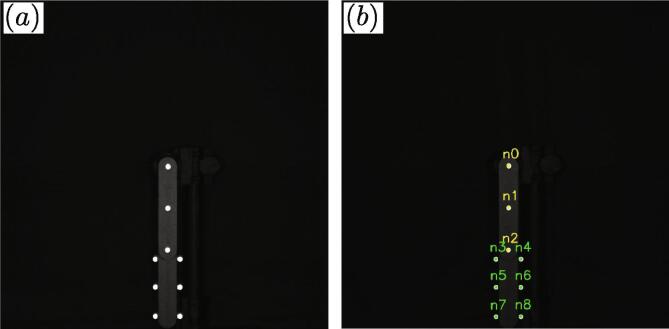
Double pendulum marker function and node locating. (a) shows the high contrast locations at the markers and (b) shows the location output from using the center of the moment contours from openCV.

To analyze the double pendulum using video data, multiple point markers were used to track the angular position of each link with the marker design discussed in Section 5. In summary, the reflective nature of the markers in comparison to the surroundings (see Fig. 6 a) allows using the *moments* function from the Python computer vision software openCV, which provides the approximate center of each marker. Fig. 6 b shows the output location of each node marked with its respective node number ($n_{1},n_{2},\ldots$).

Unfortunately, keeping track of the markers through consecutive frames is not a trivial task. It is made even more difficult by the common occurrence of occlusions with the lower link swinging behind the upper link. While one could simply omit the frames where occlusions occur as done in [16], this causes for a loss in frames that can be valuable in the analysis phase and will be avoided.

Since this tracking problem involves multiple targets, occlusions, and the motion of a rigid body, we refer to it as Multiple Object Tracking (MOT) with occlusions for rigid body dynamics. One of the most common methods for MOT with occlusions is through a Kalman filter, which requires unique characteristics between each object being tracked and a physical model constraint (usually constant acceleration or velocity). Unfortunately, the double pendulum does not adhere to either of those requirements due to its non-linear behavior, which would make implementing one of these existing methods difficult. To circumvent this issue, we develop a new method using a nearest neighbor (NN) algorithm. The NN algorithm obeys the following assumptions.

1. The method needs enough separable nodes visible at all times to distinguish between missing nodes when a reappearance occurs. Therefore, the trackers must have sufficient separation, maximum visibility (or equivalently minium chance of disappearing from the frame during motion). Other than that, if the mapping *f* of the tracked nodes through the motion (or their deformation if the object is deformable, see the point below) is Lipschitz, the placement of the points is arbitrary. The affine mapping in rigid body tracking is a special case; thus, our tracking algorithm enables tracking more than just rigid body motion. Otherwise, if the mapping of the trackers due to the object motion does not preserve the neighborhood structure, the algorithm can still be used but the user must be careful to choose the locations of the trackers such that initially neighboring points remain neighbors.
2. The targets that are initially neighbors must remain neighbors throughout the motion. such that the nearest neighbor matrix remains constant during tracking. This means that in addition to rigid bodies, deformation of flexible objects can also be tracked, if this condition is satisfied.
3. The algorithm requires the subject to have dynamics in a 2D plane. However, this method could be extended to 3D with a second camera.

While we apply this tracking algorithm for the double pendulum, the algorithm is not application dependent and could be used in several fields of research.

**Fig. 7:**
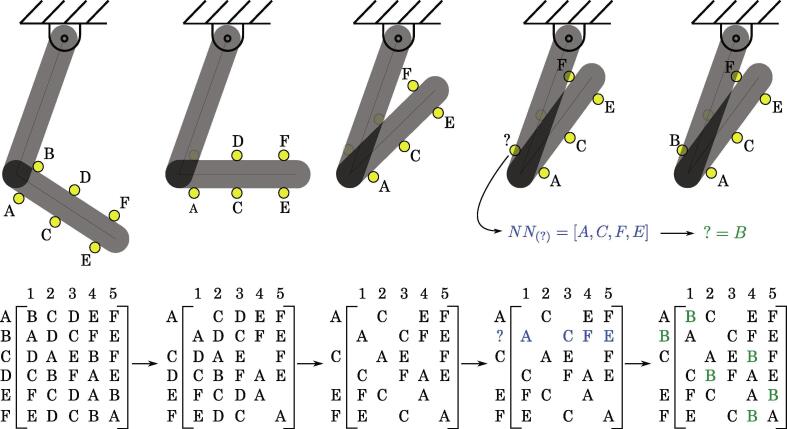
Example process of the two dimensional rigid body tracking algorithm with occlusions using a nearest neighbor scheme for the double pendulum lower link.

The tracking method first forms an NN matrix when all the nodes are visible (see left-most side of Fig. 7). Then, between frames, the first NN for all nodes in frame *t* and t-1 are used to find the updated location of each node between sequential frames. Next, if a node disappears, the NN matrix is modified to replace any reference to that node as a numpy None, and the node that disappeared is appended to a list of missing nodes (see the second configuration in Fig. 7). When a node reappears, the NNs for that node are calculated and compared to the modified NN matrix to find the best matched NN array (column in NN matrix). This reappearance procedure is shown in the two left-most configurations in Fig. 7.

The output of this tracking method is the pixel coordinates of each tracker relative to the frame over the entire video. By using MOT and this NN algorithm, one does not need to discard valuable video frames of the double pendulum’s dynamics, which is vital for accurate modeling. This tracking code is written in Python using the CV2 package and operates in approximately real-time for this application. A more detailed description of the data collection and analysis process for the tracking algorithm is provided in Appendix B. The description in Appendix B provides both an error analysis (Section B.2) and the process for translating the marker pixel coordinates to the upper and lower arm angles(Section B.3), which is built into the tracking Python script.

A common alternative to tracking the dynamics of lower pendulum arm(s) is through a wireless slip ring module [22], which introduces more mechanical complexity to the system and requires infrastructure for data transmission. By designing the double pendulum as a MOT problem, we have significantly reduced the mechanical complexity and avoided the signal transmitting problem.

The resulting video and encoder data are provided in the repository for one free drop to allow the reader to examine the data format and run the tracking Python code if desired. Additionally, encoder data and the resulting video tracking data is provided for two more free drops (no video).

### 3.4 Design files summary

.

**Table:** 

**Design filename**	**File type**	**Open source license**	**Location of the file**
Double_Pendulum_CAD.zip	SolidWorks CAD (.sldprt and.sldasm)	GNU GPL v3	Mendeley.com (see below)
simple_DAQ.m	MATLAB code (.mat)	GNU GPL v3	Mendeley.com (see below)
RBT_occ.py	Python code (.py)	GNU GPL v3	Mendeley.com (see below)
DP_free_drop_video.mp4	Free drop video (.mp4)	GNU GPL v3	Mendeley.com (see below)
Encoder_Data.zip	Encoder data (.mat files)	GNU GPL v3	Mendeley.com (see below)
Video_Tracking_Data.zip	Analyzed video data (.npy and.csv)	GNU GPL v3	Mendeley.com (see below)

Repository found at Mendeley.com and titled “Low-cost double pendulum for high-quality data collection with open-source video tracking and analysis” with DOI: 10.17632/7yd2ntbh3w.1 and link https://doi.org/10.17632/7yd2ntbh3w.1.

For each design file listed above, a short description is provided:

• Double_Pendulum_CAD.zip contains the complete SolidWorks CAD assembly for the experimental double pendulum. STL files can be easily reproduce for the 3D prtined components.
• The MATLAB code is for the national instruments data acquisition system, which allows for a user to specify the sampling rate and sampling time from a data source. We used this code to record data from the encoder attached to the upper link.
• The Python code RBT_occ.py is used to take the raw video files and track each of the markers (with occlusions in the lower link) and calculate the resulting angles from each link.
• DP_free_drop_video.mp4 is a single video of a free drop from the vertical position of the double pendulum. This video was recorded at 1000 FPS and has a size of approximately 7 GB.
• Encoder_Data.zip contains the recorded encoder voltage output as well as calibration data to translate the voltage output to an angular displacement. This data is provided for all three free drop trials.
• Video_Tracking_Data.zip contains both csv and npy (Numpy arrays) of the tracking data from the three videos as the mean angular displacement and standard deviation of the angles for both links. Additionally, a Python file is included (load_video_data.py), which is used to import the data into a Python environment.

## 4 Bill of materials

.

**Table:** 

**Designator**	**Component**	**Number**	**Cost per unit (**$**)**	**Total cost (**$**)**	**Source of materials**	**Material type**
*Post Clamp*	*Newport 340-C*	*1*	*84*	*84*	*Newport*	*Metal*
*Post Platform*	*Newport 300-P*	*1*	*217*	*217*	*Newport*	*Metal*
*Precision Rod*	*Mcmaster 6112K44*	*1*	*7*	*7*	*Mcmaster Carr*	*Metal*
*608 Bearing*	*Mcmaster 6661K12*	*2*	*9*	*18*	*Mcmaster Carr*	*Metal*
*Pan Head Cap Screw 8–32-0.5*	*Mcmaster 6661K12*	*2*	*9*	*18*	*Mcmaster Carr*	*Metal*
*Plastic Washer*	*Mcmaster 90295A310*	*1*	*7*	*7*	*Mcmaster Carr*	*Metal*
*13–20-1.5 Machine Screw*	*Mcmaster 92196A706*	*1*	*10*	*10*	*Mcmaster Carr*	*Metal*
*Slotted Spring Pin*	*Mcmaster 97161A212*	*1*	*11*	*11*	*Mcmaster Carr*	*Metal*
*E-Z LOK Insert*	*Knurled Press Insert 240*	*1*	*13*	*13*	*Grainger*	*Metal*
*Rotary Encoder*	*MCD-AVP02-0412-R060-CRW*	*1*	*200*	*200*	*Electronic*	*Posital*
*Aluminum 6061*	*Mcmaster 8975K518*	*2*	*3*	*6*	*Mcmaster Carr*	*Metal*
*Encoder Mount*	*Encoder Mount*	*1*	*–*	*–*	*Amazon*	*Polymer*
*Encoder Mount Flange*	*Encoder Flange*	*1*	*–*	*–*	*Amazon*	*Polymer*

In addition to the bill of materials necessary for constructing the double pendulum, auxiliary equipment is provided in Table 1 such as the power supply and data acquisition box that are necessary to conduct experimental measurements. These auxiliary components do not have to match exactly with the equipment used in this report, however these items should be selected so that they are compatible with one-another.

**Table 1:** Equipment used for tracking both links of double pendulum.

Description	Item Name	Manufacturer	S/N
High Speed Camera	FASTCAM Mini UX50	Photron	10445045947
Personal Computer	OptiPlex 7050	Dell	C2J7XM2
High Speed Lighting Kit	Varsa V2	Nila	V0002201
Data Acquisition System	NI USB-6356	National Instruments	1C91A61
DC Power Supply	Model 1761	BK Precision	214F16175
BNC cables	Unknown	Unknown	NA
Camera Lens	AF Zoom-NIKKOR 24–85 mm	Nikon	NA
Non-reflective Tape	Black Masking Tape	ProTapes	NA
Reflective Tape	Unknown	Unknown	NA
Backdrop	Black Background Backdrop	Issuntex	NA
Camera Stand	Unknown	Unknown	Unknown

## 5 Build instructions

The construction of the pendulum can be broken into two main categories: Base mount and pendulum links. Of the components in the bill of materials, a majority of the items may be procured through a distributor such as Mcmaster Carr. However, three items (base, upper link, and lower link) are fabricated in house. Before explaining the assembly procedure, a brief explanation of the fabrication for these parts is given.

### 5.1 Fabricated components

The most fundamental components of the double pendulum are the linkage arms. The linkages of the double pendulum are fabricated from aluminum 6061 stock which is 0.25 inches in thickness. The profile of the upper and lower links may be realized with a simple CNC milling machine capable of producing edges at 0.75 inch radius. The upper and lower links both follow this radius of curvature, however their lengths are slightly different (see Fig. 3 for details on dimensions). Three holes are machined into the upper links and two are machined into the lower link. These holes will be of use when affixing the links to one-another and to the base mount. These holes can be produced either with a CNC mill or with a standard drill press. The other fabricated components are produced with polymer 3D printing. This is a relatively straight-forward practice if one has novice experience with a 3D printing machine, and details on the 3D printed components are provided in Section 3 and are summarized in Fig. 4.

### 5.2 Assembly

**Fig. 8:**
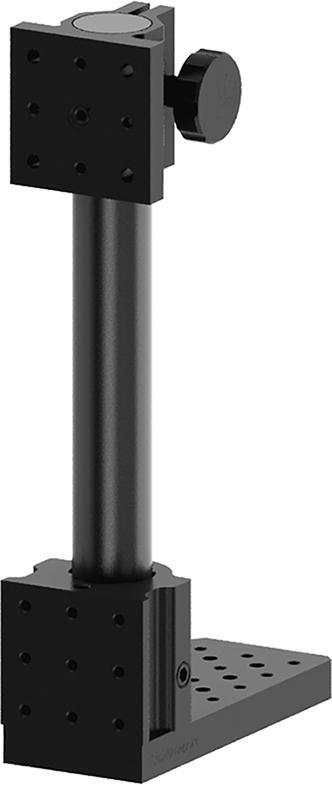
Rendering of assembled base for double pendulum.

The assembly process begins by first assembling the base mount of the pendulum. To do this, we start with a Newport 300-P optical post platform, which is secured to a bench top with four machine screws. A Newport 340-RC post clamp is then fastened to the post and it is positioned at least 15 inches above the bench top (see Fig. 8).

**Fig. 9:**
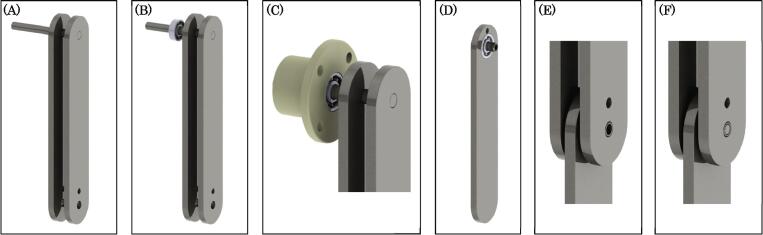
Rendering of linkage assembly process.

Next, we will describe the process for assembling the pendulum links before being connected to the encoder and 340-RC post clamp. This process begins with an 8 mm precision rod that is press fit through the top most hole of both the upper links until the rod end is flush with the outer face of the outer top link (see Fig. 9-A). The opposite rod end is then press-fit into the inner race of a 608 bearing (see Fig. 9-B). The outer race of the 608 bearing is then also press-fit into the face of the encoder mount flange (see Fig. 9-C). Next, a separate 608 bearing is press fit with the 8 mm dowel pin to its inner race, while the outer race is press fit into the top 0.197 inch diameter hole in the lower link (see Fig. 9-D). The lower link is then placed between the two upper links, and the 8 mm dowel pin is press-Fit into the 8 mm holes in the upper link (see Fig. 9-E). Next, a 5 mm detent pin is pressed into the 8 mm dowel pin to lock the dowel position in place (see Fig. 9-F). The double pendulum linkage assemble is now complete and ready to be coupled to the rotary encoder.

**Fig. 10:**
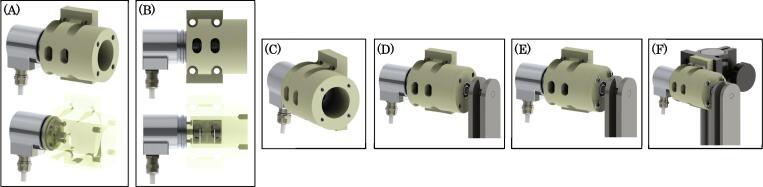
Rendering of encoder housing assembly process.

The encoder mount is first assembled separately. This construction begins by securing the rotary encoder to the mount housing with four socket head LP M3 - 0.50×8 mm screws (see Fig. 10-A). Once this is done, the shaft of the rotary encoder is connected to a 8 mm×6 mm flexible coupling which will eventually connect to the top pivot of the double pendulum (see Fig. 10-B). Next, four 8–32 E-Z LOK threaded inserts are pressed into the four 0.219 mm diameter holes on the face opposite to the encoder (see Fig. 10-C). The 8 mm precision rod which is press-fit with the inner race of the top 608 bearing is then connected to the flexible coupling to form a coupling with the encoder shaft. This action should bring the encoder mount flange to be flush with the face of the encoder mount (see Fig. 10-D). Four 8–32 pan head cap screws with plastic washers are then used to secure the encoder mount flange to the encoder mount via the four 8–32 E-Z LOK inserts (see Fig. 10-E). Lastly, the entire assembly is now mounted to the 340-RC post clamp with four 14–20×1.5 inch machine screws, thus completing the assembly (see Fig. 10-F).

**Fig. 11:**
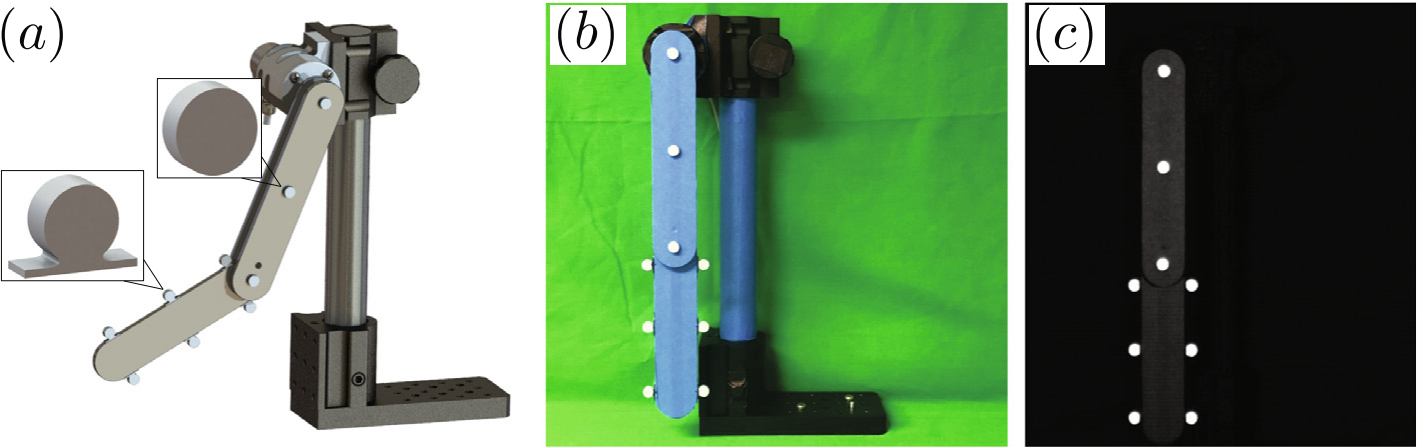
Marker design and assembly process. (a) Markers attached to double pendulum (see Section 3 for mechanical drawings), (b) physical setup with tape applied to reflective surfaces of double pendulum, and (c) initial frame of video data showing high contrast between markers and the pendulum and background.

Trackers were also be added to the double pendulum for video tracking. The two tracker types were manufactured through 3D printing at 20% infill as described in Section 3. Reflective tape is adhered to the front face for improved tracking. However, a colored tape could also be used if recording with a high speed camera with color options. These trackers are attached as shown in Fig. 11-a using a simple epoxy glue. To further improve the reflective contrast of the markers, all reflective surfaces can be covered in either a non-reflective tape and a non-reflective background can be used (see Fig. 11-b). This provides a clear contrast between other surfaces and the markers as shown in Fig. 11-c.

**Fig. 12:**
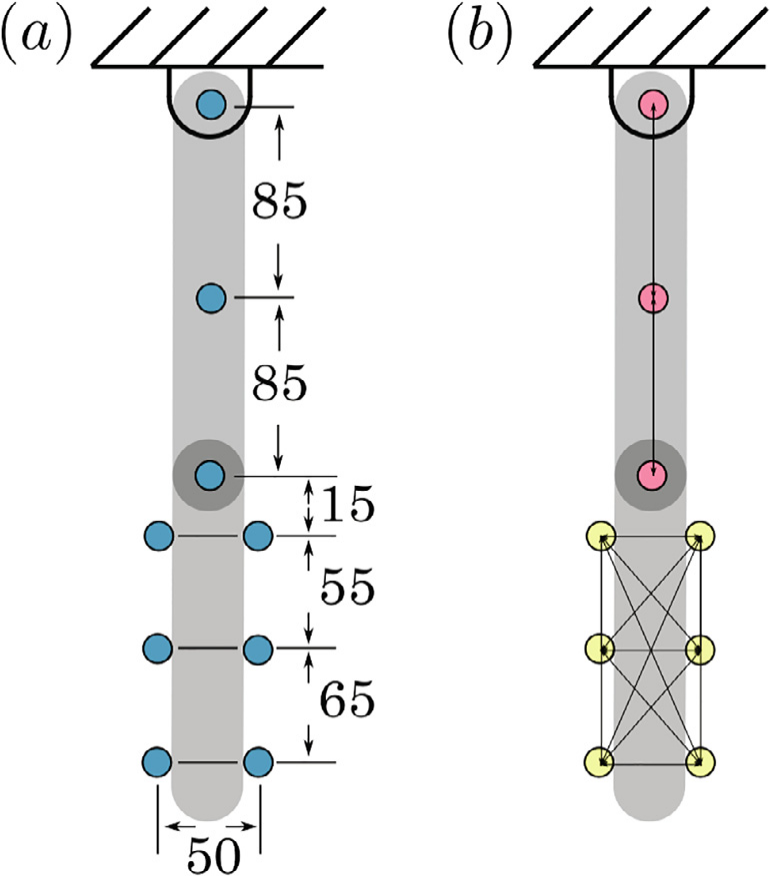
Marker locations. (a) Relative distances between markers and (b) web of relative position vectors for each rigid link.

The exact location of the markers is shown in Fig. 12-a. These locations were chosen to allow for the majority of the markers to be visible at all times. This includes when the lower link is occluded by the upper link. These locations were also chosen to allow for a large variety of position vectors between markers for each link. Specifically, a sort of “web” of relative position vectors can be formed (see Fig. 12-b). This web of relative position vectors is used to calculate $\phi_{1}$ and $\phi_{2}$ as described in Appendix B.

## 6 Operation instructions

The operating procedure for the double pendulum is relatively straightforward. However, there are multiple operating configurations which may be explored—depending on the nature of the data desired. For instance, the pendulum can be set to an initial condition manually and then released (as done for sample video and data), or it may be excited in the vertical or horizontal directions using a shaker by affixing the base of the post clamp to the base plate of a shaker. Nevertheless, this paper describes the most accessible scenario: free drops from a set initial condition.

The first task which must be performed is a calibration of the rotary encoder. The voltage output of the encoder is linearly proportional to the angle of rotation; therefore, voltage recorded from a series of known rotations can be used to construct the calibration line. A detailed example of this calibration is provided in Appendix B Section B.1.

Once the calibration is complete, the known top angle can be used to confirm that the angular measurements recorded from the encoder are commensurate with what is observed via the high speed camera and tracking algorithm. For our setup, the high speed camera is synchronized with the data acquisition system. In practice, it may be easier to trigger the camera shortly before releasing the pendulum and manually match the encoder data to the pendulum video tracking output. The tracking algorithm uses the reference initial conditions to calibrate the angles, so it is suggested to start the pendulum in the downward configuration (which is defined to be $\theta_{2}=0$ and $\theta_{1}=0$) and then start a base excitation or free drop experiment. The angle of each link is calculated using this reference position to minimize the measurement error as described in the Appendix B Section B.2. Additionally, a detailed description on how the marker locations in each frame are used to calculate each link’s angular position is provided in the Appendix B Section B.3.

A second consideration when operating the double pendulum and recording video data is the spacing between the camera and subject. This spacing should be great enough that the lens does not distort the image if a lens distortion algorithm is not applied. For the camera and software used in this work, a built-in lens distortion correction was used.

### 6.1 Notes on safe use

Potential users of the double pendulum should take note of possible dangers in operating this experimental set up. A brief list of the possible hazards is given below to ensure that safe use is practiced.

• The chaotic motion of the double pendulum is nearly impossible to anticipate. At times, all energy in the system is transferred to motion in the bottom link. After releasing the pendulum, users should maintain a distance of at least two feet to ensure that the linkages do not inadvertently strike the user.
• The user must not stop the swinging pendulum by hand since there may be a large of amount of energy in the swinging pendulum.
• All electrical components must be properly grounded. This includes the rotary encoder, power supply to the rotary encoder, and auxiliary equipment for the high speed camera
• Power supply must be turned off when configuring the wiring of the rotary encoder to prevent unwanted shock.

## 7 Validation and characterization

This section will be broken into two parts: data validation and pendulum characterization.

### 7.1 Data validation

**Fig. 13:**
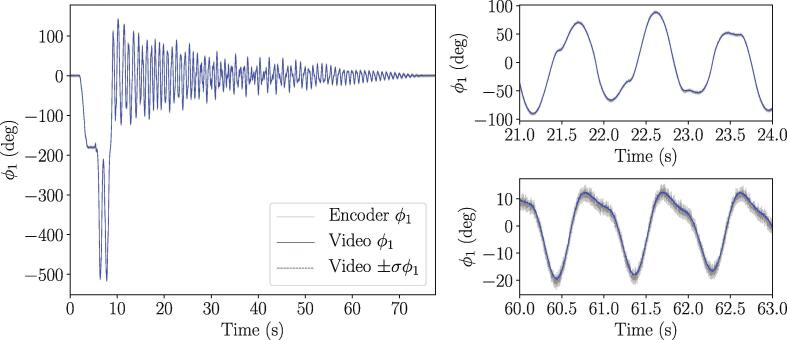
Comparison between synchronized encoder and video data of $\theta_{1}$ for validation of video data. (left) entire time series of recorded data from both encoder and video data, (top right) zoomed-in section of time series at t∈[21,24], and (bottom right) zoomed-in section of time series t∈[60,63] inclusding one standard deviation error bounds on $\theta_{1}$ which was computed using the video data.

**Fig. 14:**
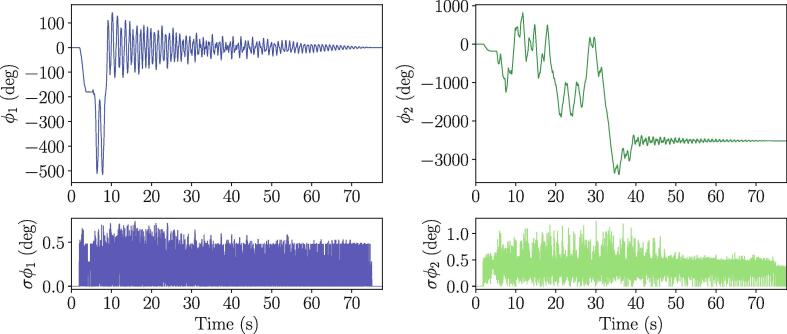
Analyzed video data for $\theta_{1}$ and $\theta_{2}$ of a free drop double pendulum with associated standard deviation of calculation for $\theta_{1}$ and $\theta_{2}$ underneath.

The tracking algorithm using the video data was validated by cross checking it with the encoder signal, where we considered the latter as the ground truth. The data was collected using free drops from the approximate initial conditions $\left[\theta_{1},{\overset{˙}{\theta }}_{2},\theta_{2},{\overset{˙}{\theta }}_{2}]=[180{}^{\circ},0,180{}^{\circ},0\right]$ at a sampling rate of 1000 Hz. However, before the comparison could be made, the video and encoder data needed to be synchronized. To do this, we implemented the synchronization software provided by Photron implemented in Photron FASTCAM Viewer 4 or PFV4. Specifically, the add-on DAQ control was used to synchronize the video data to the input channels, including the encoder, on the DAQ. This add-on allows for each frame to be associated with each recording from the DAQ, thus ensuring a direct correspondence between the encoder and the video data. Fig. 13 shows the encoder data overlaid with the video data including two zoomed in sections. The left-hand side of Fig. 13 shows that the encoder and video data are synchronized and have very similar appearance. To better examine the differences between the two data sources, the top and bottom panels on the right side of Fig. 13 further show that the amplitude and synchronization agree very well between the two data sources. Additionally, the bottom right figure shows that the tracking algorithm leads to tight error bounds. This demonstrates that the tracking algorithm is an accurate method for capturing both $\theta_{1}$ and $\theta_{2}$ for the double pendulum. To further demonstrate the tracking accuracy, Fig. 14 shows both the mean values of $\theta_{1}$ and $\theta_{2}$ as well as their one standard deviation uncertainties denoted by $\sigma \theta_{1}$ and $\sigma \theta_{2}$, respectively. Additionally, our comparisons with simulated data strongly, albeit not fully, validate the accuracy of tracking $\theta_{2}$. It should also be mentioned that the encoder has a (CPR) value of 1000, which corresponds to an accuracy of ±0.36°.

The angular velocity of two pendulum arms were estimated using the finite difference between the angular positions with the change in time know from the constant sampling rate of 1000 Hz.

Now that we have validated the tracking algorithm, the next section characterizes the damping mechanisms and determines suitable damping parameter values based on an energy loss approach from a free drop.

### 7.2 Pendulum characterization

The majority of the pendulum parameters were estimated using SolidWorks or through a direct physical measurement. A list of all the measurable component parameters are listed in Table 6 of the Appendix A.

To estimate the optimum damping parameters for the double pendulum simulation, three models were investigated. These models were viscous damping in the bearings, quadratic damping from fluid dynamics, and Coulomb damping in the bearings. These damping models are summarized in Table 2 as moments impeding motion on the upper and lower links respectively.

**Table 2:** Non-conservative damping models used for estimating damping in experimental double pendulum.

Damping Model	Damping Moment	Energy Loss
Viscous	$M_{v}=\mu_{v}\overset{˙}{\phi }$	$E_{v}=\int_{0}^{t}M_{v}\overset{˙}{\phi }\mathrm{dt}$
Quadratic	$M_{q}=\mu_{q}|\overset{˙}{\phi }|\overset{˙}{\phi }$	$E_{q}=\int_{0}^{t}M_{q}\overset{˙}{\phi }\mathrm{dt}$
Coulomb	$M_{c}=\mu_{c}\mathrm{Nr}\;\mathrm{sgn}(\overset{˙}{\phi })$	$E_{c}=\int_{0}^{t}M_{c}\overset{˙}{\phi }\mathrm{dt}$

**Fig. 15:**
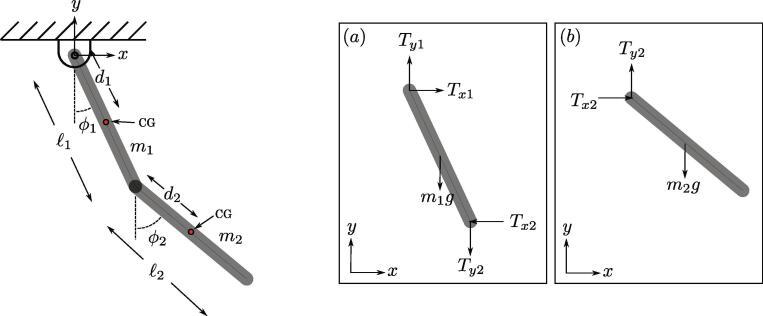
Reduced double pendulum model for two simple, symmetric links and the free body diagrams for the (a) upper and (b) lower links.

While the viscous and quadratic energy losses can be calculated using the collected data for $\phi_{1}$ and $\phi_{2}$, the Coulomb damping expression requires the normal force associated with each joint in the double pendulum. To do this, it is assumed that the double pendulum can be effectively reduced to the simple two-link double pendulum shown in Fig. 15. This assumption should hold true as all of the components of the experimental double pendulum are symmetric about the link and are, approximately, rigidly attached to each link separately. The only exception are the balls in the lower bearing, which move with respect to both the upper and the lower links—see Appendix A Section A.1 for the kinematic analysis of ball bearings. However, due to the inconsequential influence of the small bearing ball masses, we dropped them from the derivation of the normal forces. The joint normal force derivation begins by separately applying Newton’s law of motion to the upper and lower links along the *x* and *y* directions, see Fig. 15 for the corresponding Free Body Diagram (FBD). The resulting equations are

(1) $$\begin{array}{c} m_{1}{\overset{\to }{a}}_{x_{1}}=T_{x_{1}}-T_{x_{2}}\\ m_{1}{\overset{\to }{a}}_{y_{1}}=T_{y_{1}}-T_{y_{2}}-m_{1}g\\ m_{2}{\overset{\to }{a}}_{x_{2}}=T_{x_{2}}\\ m_{2}{\overset{\to }{a}}_{y_{2}}=T_{y_{2}}-m_{2}g \end{array}$$

The acceleration of the Center of Gravity (CG) of each link is calculated by taking the second time derivative of their positions given by $p_{1}=[d_{1}\sin(\phi_{1}),-d_{1}\cos(\phi_{1})]$ and $p_{2}=[\ell_{1}\sin(\phi_{1})+d_{2}\sin(\phi_{2}),-\ell_{1}\cos(\phi_{1})-d_{2}\cos(\phi_{2})]$ for the upper and lower links, respectively. This results in mass center accelerations

(2) $$\begin{array}{c} {\overset{\to }{a}}_{x_{1}}={\overset{¨}{\phi }}_{1}d_{1}\cos\left(\phi_{1}\right)-{\overset{˙}{\phi }}_{1}^{2}d_{1}\sin\left(\phi_{1}\right)\\ {\overset{\to }{a}}_{y_{1}}={\overset{¨}{\phi }}_{1}d_{1}\sin\left(\phi_{1}\right)+{\overset{˙}{\phi }}_{1}^{2}d_{1}\cos\left(\phi_{1}\right)\\ {\overset{\to }{a}}_{x_{2}}={\overset{¨}{\phi }}_{1}\ell_{1}\cos\left(\phi_{1}\right)+{\overset{¨}{\phi }}_{2}d_{2}\cos\left(\phi_{2}\right)-{\overset{˙}{\phi }}_{1}^{2}\ell_{1}\sin\left(\phi_{1}\right)-{\overset{˙}{\phi }}_{2}^{2}d_{2}\sin\left(\phi_{2}\right)\\ {\overset{\to }{a}}_{y_{2}}={\overset{¨}{\phi }}_{1}\ell_{1}\sin\left(\phi_{1}\right)+{\overset{¨}{\phi }}_{2}d_{2}\sin\left(\phi_{2}\right)+{\overset{˙}{\phi }}_{1}^{2}\ell_{1}\cos\left(\phi_{1}\right)+{\overset{˙}{\phi }}_{2}^{2}d_{2}\cos\left(\phi_{2}\right) \end{array}$$

Next, the reaction terms in the upper and lower joints $\left(T_{x1},T_{y1},T_{x2},T_{y2}\right)$ can be resolved by applying Newton’s law of motion in the *x* and *y* directions for both the upper and lower links as shown in Eq. (1). Using these reactions, the magnitude of the normal force was then calculated as $N_{i}=\sqrt{T_{x_{i}}^{2}+T_{y_{i}}^{2}}$ for both links, resulting in the simplified normal forces

(3) $$\begin{array}{c} N_{1}={\left[d_{1}m_{1}\left(-\overset{¨}{\phi_{1}}\cos\left(\phi_{1}\right)+{\overset{˙}{\phi_{1}}}^{2}\sin\left(\phi_{1}\right)\right)+m_{2}\left(-\overset{¨}{\phi_{1}}\ell_{1}\cos\left(\phi_{1}\right)-\overset{¨}{\phi_{2}}d_{2}\cos\left(\phi_{2}\right)+{\overset{˙}{\phi_{1}}}^{2}\ell_{1}\sin\left(\phi_{1}\right)+{\overset{˙}{\phi_{2}}}^{2}d_{2}\sin\left(\phi_{2}\right)\right)\right]}^{2}+{\left[d_{1}m_{1}\left(\overset{¨}{\phi_{1}}\sin\left(\phi_{1}\right)+{\overset{˙}{\phi_{1}}}^{2}\cos\left(\phi_{1}\right)\right)+g(m_{1}+m_{2})+m_{2}\left(\overset{¨}{\phi_{1}}\ell_{1}\sin\left(\phi_{1}\right)+\overset{¨}{\phi_{2}}d_{2}\sin\left(\phi_{2}\right)+{\overset{˙}{\phi_{1}}}^{2}\ell_{1}\cos\left(\phi_{1}\right)+{\overset{˙}{\phi_{2}}}^{2}d_{2}\cos\left(\phi_{2}\right)\right)\right]}^{2},\\ N_{2}=m_{2}^{2}{\left({\overset{¨}{\phi }}_{1}\ell_{1}\cos\left(\phi_{1}\right)+{\overset{¨}{\phi }}_{2}d_{2}\cos\left(\phi_{2}\right)-{\overset{˙}{\phi }}_{1}^{2}\ell_{1}\sin\left(\phi_{1}\right)-{\overset{˙}{\phi }}_{2}^{2}d_{2}\sin\left(\phi_{2}\right)\right)}^{2}+m_{2}^{2}{\left({\overset{¨}{\phi }}_{1}\ell_{1}\sin\left(\phi_{1}\right)+{\overset{¨}{\phi }}_{2}d_{2}\sin\left(\phi_{2}\right)+{\overset{˙}{\phi }}_{1}^{2}\ell_{1}\cos\left(\phi_{1}\right)+{\overset{˙}{\phi }}_{2}^{2}d_{2}\cos\left(\phi_{2}\right)+g\right)}^{2}, \end{array}$$

where the other (non-damping) summary parameters used in this calculated are provided in Table 4 of the Appendix A, which were estimated using SolidWorks. By substituting these normal forces into the Coulomb damping expression in Table 2, the energy loss is expressed as

(4) $$\begin{array}{c} E_{c}^{\left(1\right)}=\int_{0}^{t}\mu_{c}^{\left(1\right)}N_{1}r\mathrm{sgn}(\overset{˙}{\phi_{1}})\overset{˙}{\phi_{1}}\mathrm{dt},\\ E_{c}^{\left(2\right)}=\int_{0}^{t}\mu_{c}^{\left(2\right)}N_{2}r\mathrm{sgn}(\overset{˙}{\phi_{2}}-\overset{˙}{\phi_{1}})(\overset{˙}{\phi_{2}}-\overset{˙}{\phi_{1}})\mathrm{dt}, \end{array}$$

where r≈0.008 m as the radius of the upper and lower shafts and coulomb damping constant $\mu_{c}^{i}$ with the superscript denoting which joint/link the constant or energy loss belongs to, respectively. The energy loss for viscous damping is calculated as

(5) $$\begin{array}{c} E_{v}^{\left(1\right)}=\int_{0}^{t}\mu_{v}^{\left(1\right)}{\overset{˙}{\phi }}_{1}^{2}\mathrm{dt},\\ E_{v}^{\left(2\right)}=\int_{0}^{t}\mu_{v}^{\left(2\right)}{\left({\overset{˙}{\phi }}_{2}-{\overset{˙}{\phi }}_{1}\right)}^{2}\mathrm{dt}, \end{array}$$

where $\mu_{v}^{\left(1\right)}$ and $\mu_{v}^{\left(2\right)}$ are the viscous damping constants for the upper and lower links, respectively. Lastly, the energy loss for Quadratic damping is calculated as

(6) $$\begin{array}{c} E_{q}^{\left(1\right)}=\int_{0}^{t}\mu_{q}^{\left(1\right)}|{\overset{˙}{\phi }}_{1}|{\overset{˙}{\phi }}_{1}^{2}\mathrm{dt},\\ E_{q}^{\left(2\right)}=\int_{0}^{t}\mu_{q}^{\left(2\right)}|{\overset{˙}{\phi }}_{2}|{\overset{˙}{\phi }}_{2}^{2}\mathrm{dt}, \end{array}$$

where $\mu_{q}^{\left(1\right)}$ and $\mu_{q}^{\left(2\right)}$ are the quadratic damping constants for the upper and lower links, respectively.

To calculate the actual energy profile of the double pendulum, the energy of each component was considered. For a detailed description of the energy of each component please reference Section A in Appendix A.

**Fig. 16:**
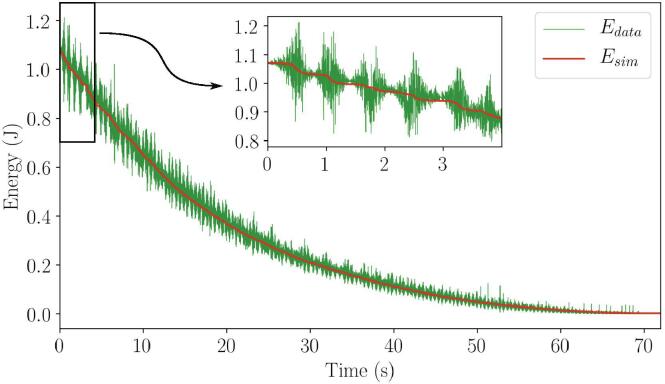
Zoomed-in section of the energy profile between experimental and simulated energy loss at beginning of drop.

**Fig. 17:**
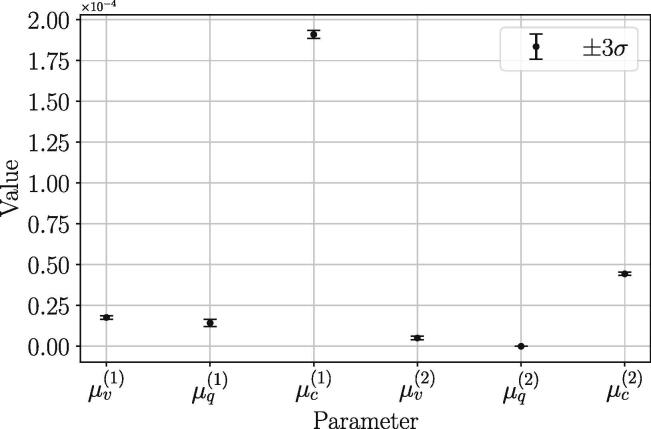
Damping parameter optimization results for three trials of the free-drop double pendulum.

Using the energy loss Eqs. (4), (5), (6), the energy profile was optimized to fit the actual energy profile as shown in Fig. 16. Additionally, a zoomed-in section of the beginning of the energy profile in Fig. 16 shows a general matching between the simulated energy and energy from the data. A possible cause for the imperfect matching of the two energy profiles may be due to the inherent noise in the data collected, which has a more severe effect on the simulated energy profile, as it is dependent on the second time derivative of the angles. The simulated energy profile was matched by optimizing the damping constants for each damping model using the BFGS optimization method. This optimization was done for three trials. The resulting parameter values with their associated uncertainty are shown in Fig. 17.

**Fig. 18:**
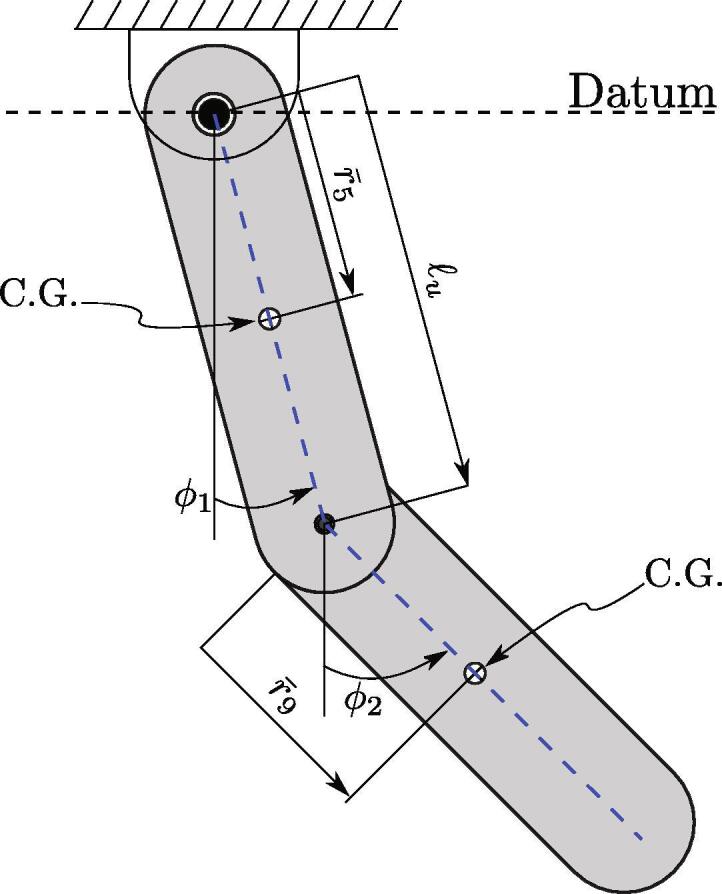
Double pendulum reference angles and datum.

**Fig. 19:**
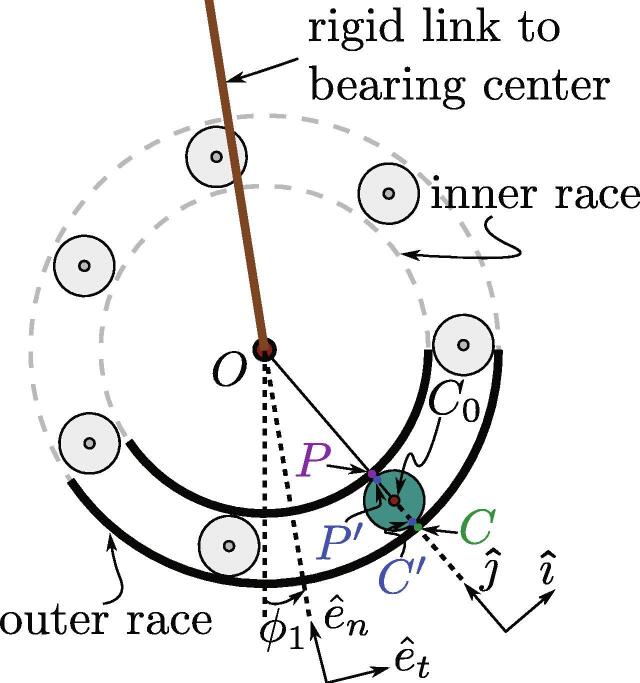
Schematic of the ball bearing used (the retainer ring is not shown).

As shown in Table 3, the magnitudes of the damping coefficients are heavily weighted towards Coulomb damping. This weighting was validated by calculating the percent of the total energy dissipated by each damping method. We found that viscous, quadratic and Coulomb damping account for approximately 9%,22%, and 69% of the total energy loss, respectively. This concludes that the majority of the energy dissipation is from Coulomb damping in the bearings. Additionally, we calculated that approximately 81% of the energy was dissipated through the upper link and bearing, while only 19% through the lower link and bearing. This result suggests that the encoder responsible for significant energy loss and damping. If it is desired to further reduce the damping effects on the dynamics, it is suggested to detach the encoder and only track the double pendulum using the video tracking algorithm (Fig. 18, Fig. 19).

**Table 3:** Optimized damping parameters with associated uncertainty (one standard deviation) from three trials.

Parameter	Value	Uncertainty (±σ)
$\mu_{v}^{\left(1\right)}$	1.76E-05	3.42E-07
$\mu_{q}^{\left(1\right)}$	1.43E-05	7.24E-07
$\mu_{c}^{\left(1\right)}$	1.91E-04	6.05E-07
$\mu_{v}^{\left(2\right)}$	5.08E-06	3.89E-07
$\mu_{q}^{\left(2\right)}$	0.00E + 00	0.00E + 00
$\mu_{c}^{\left(2\right)}$	4.44E-05	3.46E-07

**Table 4:** Approximate Simplified double pendulum parameters (see Fig. 15).

Parameter	Value
$m_{1}$	0.311 kg
$m_{2}$	0.111 kg
$d_{1}$	0.079 m
$d_{2}$	0.071 m
$\ell_{1}$	0.172 m
$\ell_{2}$	0.143 m

We have also provided a complete derivation of the full equations of motion for this experimental design in Appendix A using Lagrange’s equation. This facilitates removing elements of the design (e.g. the encoder) from the equations of motion.

## Human and animal rights

No human or animal subjects were used in this experiment.

## Declaration of Competing Interest

The authors declare that they have no known competing financial interests or personal relationships that could have appeared to influence the work reported in this paper.

## Figures and Tables

**Fig. 20:**
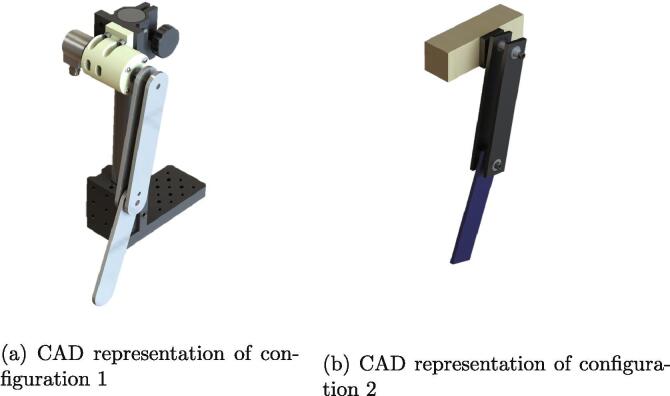
Two possible configurations for the double pendulum. The left configuration is the one we used in this paper, which has fewer bearings than the configuration on the right. See Table 5 for the corresponding bearing angular velocity expressions.

**Table 5:** Angular velocity expressions for the two bearing configurations shown in Fig. 20. Configuration one is the one used in this paper.

	configuration 1	configuration 2
$\omega_{C}$	$\frac{d_{I}}{2d_{B}}{\overset{˙}{\phi }}_{1}-\frac{\left(d_{I}+2d_{B}\right)}{2d_{B}}{\overset{˙}{\phi }}_{2}$	$\frac{d_{I}}{2d_{B}}{\overset{˙}{\phi }}_{2}-\frac{\left(d_{I}+2d_{B}\right)}{2d_{B}}{\overset{˙}{\phi }}_{1}$

$\omega_{D}$	$\frac{d_{I}}{2\left(d_{I}+d_{B}\right)}{\overset{˙}{\phi }}_{1}+\frac{d_{I}+2d_{B}}{2\left(d_{I}+d_{B}\right)}{\overset{˙}{\phi }}_{2}$	$\frac{d_{I}}{2\left(d_{I}+d_{B}\right)}{\overset{˙}{\phi }}_{2}+\frac{d_{I}+2d_{B}}{2\left(d_{I}+d_{B}\right)}{\overset{˙}{\phi }}_{1}$

**Fig. 21:**
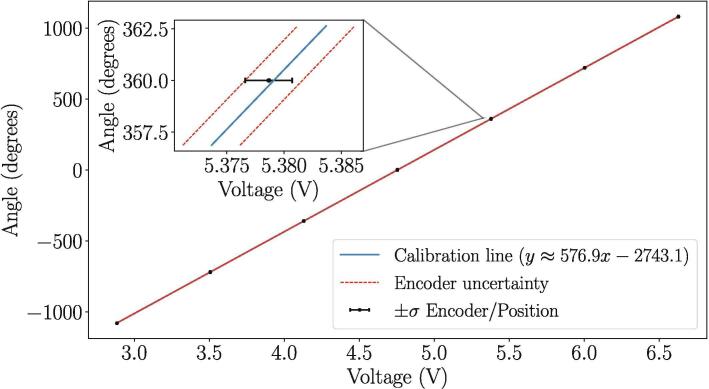
Calibration line for the encoder used to measure the angle of the top link of the double pendulum.

**Fig. 22:**
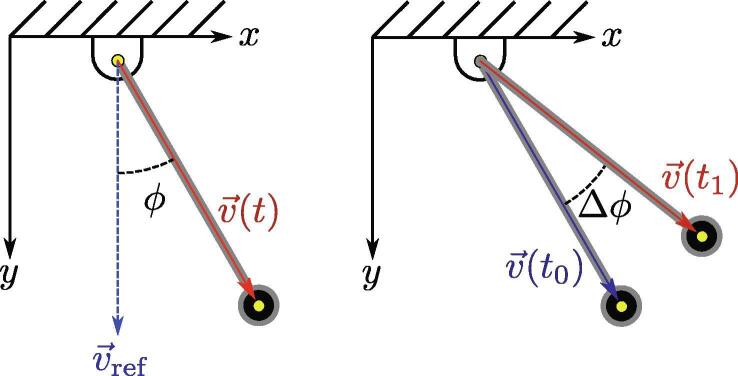
Visual representation of two methods for calculating ϕ using video data: method 1 on the left and method 2 on the right.

**Fig. 23:**
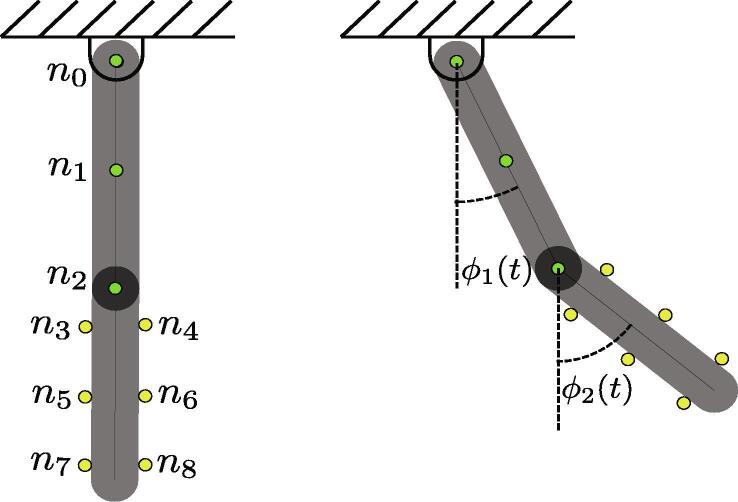
Double pendulum diagram showing node assignment on the left and $\phi_{1}$ and $\phi_{2}$ reference on the right.

**Fig. 24:**
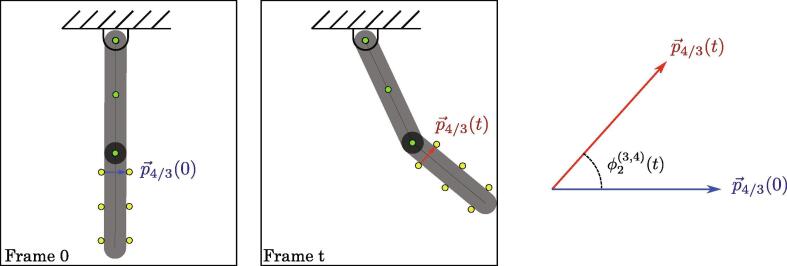
Example showing how $\phi_{2}(t)$ is found using the original down configuration and a later configuration at time *t* for the vector between nodes $n_{3}$ and $n_{4}$.

**Table 6:** Values and uncertainties of system variables

**Variable**	**Description**	**Obtained with**	**Value**	**Unit**
$m_{1}$	Encoder mass	Not applicable	*NA*	[g]
$m_{2}$	Flex coupling	SP601 balance	22.3±0.1	[g]
$m_{3,1}$	Bearing inner race	SP601 balance	2.9±0.1	[g]
$m_{3,2}$	Bearing ball spacer	SP601 balance	0.2±0.1	[g]
$m_{3,3}$	Bearing ball	SP601 balance	0.3±0.1	[g]
$m_{4}$	Upper shaft	SP601 balance	30±0.5	[g]
$m_{5}$	Upper arm	Calculation	132±2	[g]
$m_{6}$	Lower shaft	Calculation	3.8±0.2	[g]
$m_{7}$	Dowel pin	Manufact. spec.	2.7±0.2	[g]
$m_{8,1}$	Bearing inner race	SP601 balance	2.9±0.1	[g]
$m_{8,2}$	Bearing ball spacer	SP601 balance	0.2±0.1	[g]
$m_{8,3}$	Bearing ball	SP601 balance	0.3±0.1	[g]
$m_{8,4}$	Bearing outer race	SP601 balance	6.5±0.1	[g]
$m_{9}$	Lower arm	Calculation	116±2	[g]
$m_{10}$	Upper tracker	SP601 balance	0.2±0.1	[g]
$m_{11}$	Lower tracker	SP601 balance	0.3±0.1	[g]
$m_{12}$	Upper arm tape	SP601 balance	0.7±0.1	[g]
$m_{13}$	Lower arm tape	SP601 balance	0.6±0.1	[g]
${\overset{¯}{r}}_{4}$	Top pivot to set screw	Calculation	4.0±0.5	[mm]
$\ell_{u}$	Top to bottom pivot	Calculation	171.9±0.3	[mm]
${\overset{¯}{r}}_{5}$	Top pivot to bar 1.	Calculation	86.0±0.2	[mm]
${\overset{¯}{r}}_{9}$	Bottom pivot to bar 2	Calculation	71.5±0.4	[mm]
${\overset{‾}{I}}_{1}$	Encoder inertia	Manufact. spec.	2000±200	$\left[g\cdot {\mathrm{mm}}^{2}\right]$
${\overset{‾}{I}}_{2}$	Flex couple inertia	SolidWorks	1100±5	$\left[g\cdot {\mathrm{mm}}^{2}\right]$
${\overset{‾}{I}}_{3,1}$	Inner race inertia	SolidWorks	103	$\left[g\cdot {\mathrm{mm}}^{2}\right]$
${\overset{‾}{I}}_{3,2}$	Ball spacer inertia	Calculated	40±20	$\left[g\cdot {\mathrm{mm}}^{2}\right]$
${\overset{‾}{I}}_{3,3}$	Bearing ball inertia	SolidWorks	0.4	$\left[g\cdot {\mathrm{mm}}^{2}\right]$
${\overset{‾}{I}}_{4}$	Upper shaft inertia	Calculated	242±4	$\left[g\cdot {\mathrm{mm}}^{2}\right]$
${\overset{‾}{I}}_{5}$	Upper arm inertia	Calculated	470000±9000	$\left[g\cdot {\mathrm{mm}}^{2}\right]$
${\overset{‾}{I}}_{6}$	Lower shaft inertia	Calculated	12±1	$\left[g\cdot {\mathrm{mm}}^{2}\right]$
${\overset{‾}{I}}_{7}$	Dowel pin inertia	Calculated	61±2	$\left[g\cdot {\mathrm{mm}}^{2}\right]$
${\overset{‾}{I}}_{8,1}$	Inner race inertia	SolidWorks	103	$\left[g\cdot {\mathrm{mm}}^{2}\right]$
${\overset{‾}{I}}_{8,2}$	Ball spacer inertia	Calculated	2.5	$\left[g\cdot {\mathrm{mm}}^{2}\right]$
${\overset{‾}{I}}_{8,3}$	Bearing ball inertia	SolidWorks	0.4	$\left[g\cdot {\mathrm{mm}}^{2}\right]$
${\overset{‾}{I}}_{8,4}$	Outer race inertia	SolidWorks	544	$\left[g\cdot {\mathrm{mm}}^{2}\right]$
${\overset{‾}{I}}_{9}$	Bottom bar inertia	Calculated	245000±5000	$\left[g\cdot {\mathrm{mm}}^{2}\right]$
${\overset{‾}{I}}_{10}$	Upper tracker	SolidWorks	4±1	$\left[g\cdot {\mathrm{mm}}^{2}\right]$
${\overset{‾}{I}}_{11}$	Lower tracker	SolidWorks	9±1	$\left[g\cdot {\mathrm{mm}}^{2}\right]$
${\overset{‾}{I}}_{12}$	Upper arm tape	SolidWorks	2500±200	$\left[g\cdot {\mathrm{mm}}^{2}\right]$
${\overset{‾}{I}}_{13}$	Lower arm tape	SolidWorks	1300±200	$\left[g\cdot {\mathrm{mm}}^{2}\right]$
$d_{I}$	Inner race diameter	Calipers	11.062±0.002	[mm]
$d_{B}$	Bearing ball diameter	Calipers	3.932±0.002	[mm]

## Appendix A Equations of motion and model parameters

Lagrange’s equation is used to develop the equations of motion for the experimental double pendulum. Consequently, the kinetic and potential energy of every moving component must first be determined. The moving elements include the following: the encoder, upper bearing, flexible coupling, upper shaft, upper links, upper trackers, lower bearing, hollow dowel pin, lower shaft, lower link, lower trackers, and optional non-reflective tape for the links.

1. Encoder (x1):
  • Mass: $m_{1}$, Inertia: $I_{1}={\overset{‾}{I}}_{1}$, Center of Mass: $X_{1}=0,Y_{1}=0$
  • Kinetic Energy: $T_{1}=\frac{1}{2}I_{1}{\overset{˙}{\phi }}_{1}^{2}$
  • Potential Energy: $V_{1}=0$
2. Flexible Coupling (x1):
  • Mass: $m_{2}$, Inertia: $I_{1}={\overset{‾}{I}}_{1}$, Center of Mass: $X_{2}=0,Y_{2}=0$
  • Kinetic Energy: $T_{2}=\frac{1}{2}I_{2}{\overset{˙}{\phi }}_{1}^{2}$
  • Potential Energy: $V_{2}=0$
3. Upper Bearing (x1): Reference Section A.1
  (a) Inner Race (x1):
    • Mass: $m_{3,1}$, Inertia: $I_{3,1}={\overset{‾}{I}}_{3,1}$, Center of Mass: $X_{3,1}=0,Y_{3,1}=0$
    • Kinetic Energy: $T_{3,1}=\frac{1}{2}I_{3,1}{\overset{˙}{\phi }}_{1}^{2}$
    • Potential Energy: $V_{3,1}=0$
  (b) Ball Spacer (x1):
    • Mass: $m_{3,2}$, Inertia: $I_{3,2}={\overset{‾}{I}}_{3,2}$, Center of Mass: $X_{3,2}=0,Y_{3,2}=0$
    • Kinetic Energy: $T_{3,2}=\frac{1}{2}I_{3,2}{\left(\frac{d_{I}}{2(d_{I}+d_{B})}{\overset{˙}{\phi }}_{1}\right)}^{2}$
    • Potential Energy: $V_{3,2}=0$
  (c) Bearing Ball (x7):
    • Mass: $m_{3,3}$, Inertia: $I_{3,3}={\overset{‾}{I}}_{3,3}$, Center of Mass: $X_{3,3}=0,Y_{3,3}=0$.
    • Kinetic Energy: $T_{3,3}=\frac{1}{2}m_{3,3}{\left(\frac{d_{I}}{4}{\overset{˙}{\phi }}_{1}\right)}^{2}+\frac{1}{2}I_{3,3}{\left(\frac{d_{I}}{2d_{B}}{\overset{˙}{\phi }}_{1}\right)}^{2}$
    • Potential Energy (Center of Ball Set Equals Zero): $V_{3,3}=0$
4. Upper Shaft (x1):
  • Mass: $m_{4}$, Inertia: $I_{4}={\overset{‾}{I}}_{4}$.
  • Kinetic Energy: $T_{4}=\frac{1}{2}I_{4}{\overset{˙}{\phi }}_{1}^{2}$
  • Potential Energy: $V_{4}=m_{4}g{\overset{¯}{r}}_{4}(1-\cos\phi_{1})$
5. Upper Arm (x2):
  • Mass: $m_{5}$, Inertia: $I_{5}={\overset{‾}{I}}_{5}+m_{5}{\overset{¯}{r}}_{5}^{2}$, Center of Mass: $X_{5}={\overset{¯}{r}}_{5}\sin\phi_{1},Y_{5}=-{\overset{¯}{r}}_{5}\cos\phi_{1}$
  • Kinetic Energy: $T_{5}=\frac{1}{2}I_{5}{\overset{˙}{\phi }}_{1}^{2}$
  • Potential Energy: $V_{5}=m_{5}g{\overset{¯}{r}}_{5}(1-\cos\phi_{1})$
6. Lower Shaft (x1):
  • Mass: $m_{6}$, Inertia: $I_{6}={\overset{‾}{I}}_{6}+m_{6}\ell_{u}^{2}$, Center of Mass: $X_{6}=\ell_{u}\sin\phi_{1},Y_{6}=-\ell_{u}\cos\phi_{1}$
  • Kinetic Energy: $T_{6}=\frac{1}{2}I_{6}{\overset{˙}{\phi }}_{1}^{2}$
  • Potential Energy: $V_{6}=m_{6}g\ell_{u}\left(1-\cos\phi_{1}\right)$
7. Dowel Pin (x1):
  • Mass: $m_{7}$, Inertia: $I_{7}={\overset{‾}{I}}_{7}+m_{7}\ell_{u}^{2}$, Center of Mass: $X_{7}=\ell_{u}\sin\phi_{1},Y_{7}=-\ell_{u}\cos\phi_{1}$
  • Kinetic Energy: $T_{7}=\frac{1}{2}I_{7}{\overset{˙}{\phi }}_{1}^{2}$
  • Potential Energy: $V_{7}=m_{7}g\ell_{u}\left(1-\cos\phi_{1}\right)$
8. Lower Bearing (x1): Reference Section A.1
  (a) Inner Race (x1):
    • Mass: $m_{8,1}$, Inertia: $I_{8,1}={\overset{‾}{I}}_{8,1}+m_{8,1}\ell_{u}^{2}$, Center of Mass: $X_{8,1}=\ell_{u}\sin\phi_{1},Y_{8,1}=-\ell_{u}\cos\phi_{1}$
    • Kinetic Energy: $T_{8,1}=\frac{1}{2}I_{8,1}{\overset{˙}{\phi }}_{1}^{2}$
    • Potential Energy: $V_{8,1}=m_{8,1}g\ell_{u}\left(1-\cos\phi_{1}\right)$
  (b) Ball Spacer (x1):
    • Mass: $m_{8,2}$, Inertia: $I_{8,2}={\overset{‾}{I}}_{8,2}+m_{8,2}\ell_{u}^{2}$, Center of Mass: $X_{8,2}=\ell_{u}\sin\phi_{1},Y_{8,2}=-\ell_{u}\cos\phi_{1}$
    • Kinetic Energy: $T_{8,2}=\frac{1}{2}m_{8,2}{\left(\ell_{u}{\overset{˙}{\phi }}_{1}\right)}^{2}+\frac{1}{2}{\overset{‾}{I}}_{8,2}{\left(\frac{d_{I}}{2\left(d_{I}+d_{B}\right)}{\overset{˙}{\phi }}_{1}+\frac{d_{1}+2d_{B}}{2\left(d_{I}+d_{B}\right)}{\overset{˙}{\phi }}_{2}\right)}^{2}$
    • Potential Energy: $V_{8,2}=m_{8,2}g\ell_{u}\left(1-\cos\phi_{1}\right)$
  (c) Bearing Balls (x7):
    • Mass: $m_{8,3}$, Inertia: $I_{8,3}={\overset{‾}{I}}_{8,3}+m_{8,3}\ell_{u}^{2}$, Center of Mass: $X_{8,3}=\ell_{u}\sin\phi_{1},Y_{8,3}=-\ell_{u}\cos\phi_{1}$. Note: This expression accounts for all 7 bearing balls.
    • Kinetic Energy: $T_{8,3}=\frac{7}{2}m_{8,3}\left[{\left(\ell_{u}{\overset{˙}{\phi }}_{1}\right)}^{2}+{\left(\frac{d_{I}}{4}\overset{˙}{\phi_{1}}+\frac{d_{I}+2d_{B}}{4}{\overset{˙}{\phi }}_{2}\right)}^{2}\left(\ell_{u}{\overset{˙}{\phi }}_{1}\right)\left(\frac{d_{I}}{2(d_{I}+d_{B})}\overset{˙}{\phi_{1}}+\frac{d_{I}+2d_{B}}{2(d_{I}+d_{B})}{\overset{˙}{\phi }}_{2}\right)\left(d_{I}+d_{B}\right)\sum_{i=1}^{7}\cos\left(\frac{2\pi (i-1)}{7}+(\theta_{i}-\phi_{1})\right)\right]+\frac{7}{2}I_{8,3}{\left(\frac{d_{I}}{2d_{B}}{\overset{˙}{\phi }}_{1}-\frac{\left(d_{I}+2d_{B}\right)}{2d_{B}}{\overset{˙}{\phi }}_{2}\right)}^{2}$
    • Potential Energy (Center of Ball Set): $V_{8,3}=7m_{8,3}g\ell_{u}\left(1-\cos\phi_{1}\right)$
  (d) Outer Race (x1):
    • Mass: $m_{8,4}$, Inertia: $I_{8,4}={\overset{‾}{I}}_{8,4}+m_{8,4}\ell_{u}^{2}$, Center of Mass: $X_{8,4}=\ell_{u}\sin\phi_{1},Y_{8,4}=-\ell_{u}\cos\phi_{1}$
    • Kinetic Energy: $T_{8,4}=\frac{1}{2}m_{8,4}{\left(\ell_{u}{\overset{˙}{\phi }}_{1}\right)}^{2}+\frac{1}{2}{\overset{‾}{I}}_{8,4}{\overset{˙}{\phi }}_{2}^{2}$
    • Potential Energy: $V_{8,4}=m_{8,4}g\ell_{u}\left(1-\cos\phi_{1}\right)$
9. Lower Arm (x1):
  • Mass: $m_{9}$, Inertia: $I_{9}={\overset{‾}{I}}_{9}+m_{9}\left[{\left(\ell_{u}\cos\phi_{1}+{\overset{¯}{r}}_{9}\cos\phi_{2}\right)}^{2}+{\left(\ell_{u}\sin\phi_{1}+{\overset{¯}{r}}_{9}\sin\phi_{2}\right)}^{2}\right]$, Center of Mass: $X_{9}=\ell_{u}\sin\phi_{1}+{\overset{¯}{r}}_{9}\sin\phi_{2},Y_{9}=-\ell_{u}\cos\phi_{1}-{\overset{¯}{r}}_{9}\cos\phi_{2}$
  • Kinetic Energy: $T_{9}=\frac{1}{2}m_{9}\ell_{u}^{2}{\overset{˙}{\phi }}_{1}^{2}+m_{9}\ell_{u}{\overset{¯}{r}}_{9}{\overset{˙}{\phi }}_{1}{\overset{˙}{\phi }}_{2}\cos\left(\phi_{2}-\phi_{1}\right)+\frac{1}{2}m_{9}{\overset{¯}{r}}_{9}^{2}{\overset{˙}{\phi }}_{2}^{2}+\frac{1}{2}{\overset{‾}{I}}_{9}{\overset{˙}{\phi }}_{2}^{2}$
  • Potential Energy: $V_{9}=m_{9}g\left[\ell_{u}\left(1-\cos\phi_{1}\right)+{\overset{¯}{r}}_{9}\left(1-\cos\phi_{2}\right)\right]$
10. Upper Trackers (x3):
  • Mass: $m_{10}$, Inertia: $I_{10}={\overset{‾}{I}}_{10}+m_{10}{\overset{¯}{r}}_{10,i}^{2}$, Center of Mass: $\left[X_{10,i},Y_{10,i}\right]$ is dependent on marker location relative to link.
  • Kinetic Energy: $T_{10}=\frac{1}{2}I_{10}{\overset{˙}{\phi }}_{1}^{2}$
  • Potential Energy: $V_{10}=m_{10}g{\overset{¯}{r}}_{10}(1-\cos\phi_{1})$
11. Lower Trackers (x6):
  • Mass: $m_{11}$, Inertia: $I_{11}={\overset{‾}{I}}_{11}+m_{11}\left[{\left(\ell_{u}\cos\phi_{1}+{\overset{¯}{r}}_{11}\cos\phi_{2}\right)}^{2}+{\left(\ell_{u}\sin\phi_{1}+{\overset{¯}{r}}_{11}\sin\phi_{2}\right)}^{2}\right]$, Center of Mass: $\left[X_{10,i},Y_{10,i}\right]$ is dependent on marker location relative to link.
  • Kinetic Energy: $T_{11}=\frac{1}{2}m_{11}\ell_{u}^{2}{\overset{˙}{\phi }}_{1}^{2}+m_{11}\ell_{u}{\overset{¯}{r}}_{11}{\overset{˙}{\phi }}_{1}{\overset{˙}{\phi }}_{2}\cos\left(\phi_{2}-\phi_{1}\right)+\frac{1}{2}m_{11}{\overset{¯}{r}}_{11}^{2}{\overset{˙}{\phi }}_{2}^{2}+\frac{1}{2}{\overset{‾}{I}}_{11}{\overset{˙}{\phi }}_{2}^{2}$
  • Potential Energy: $V_{11}=m_{11}g\left[\ell_{u}\left(1-\cos\phi_{1}\right)+{\overset{¯}{r}}_{11}\left(1-\cos\phi_{2}\right)\right]$
12. Upper Arm Tape (x1):
  • Mass: $m_{12}$, Inertia: $I_{12}={\overset{‾}{I}}_{12}+m_{12}{\overset{¯}{r}}_{12}^{2}$, Center of Mass: $X_{12}={\overset{¯}{r}}_{12}\sin\phi_{1},Y_{12}=-{\overset{¯}{r}}_{12}\cos\phi_{1}$
  • Kinetic Energy: $T_{12}=\frac{1}{2}I_{12}{\overset{˙}{\phi }}_{1}^{2}$
  • Potential Energy: $V_{12}=m_{12}g{\overset{¯}{r}}_{12}(1-\cos\phi_{1})$
13. Lower Arm Tape (x1):
  • Mass: $m_{13}$, Inertia: $I_{13}={\overset{‾}{I}}_{13}+m_{13}\left[{\left(\ell_{u}\cos\phi_{1}+{\overset{¯}{r}}_{13}\cos\phi_{2}\right)}^{2}+{\left(\ell_{u}\sin\phi_{1}+{\overset{¯}{r}}_{13}\sin\phi_{2}\right)}^{2}\right]$, Center of Mass: $X_{13}=\ell_{u}\sin\phi_{1}+{\overset{¯}{r}}_{13}\sin\phi_{2},Y_{13}=-\ell_{u}\cos\phi_{1}-{\overset{¯}{r}}_{13}\cos\phi_{2}$
  • Kinetic Energy: $T_{13}=\frac{1}{2}m_{13}\ell_{u}^{2}{\overset{˙}{\phi }}_{1}^{2}+m_{13}\ell_{u}{\overset{¯}{r}}_{13}{\overset{˙}{\phi }}_{1}{\overset{˙}{\phi }}_{2}\cos\left(\phi_{2}-\phi_{1}\right)+\frac{1}{2}m_{13}{\overset{¯}{r}}_{13}^{2}{\overset{˙}{\phi }}_{2}^{2}+\frac{1}{2}{\overset{‾}{I}}_{13}{\overset{˙}{\phi }}_{2}^{2}$
  • Potential Energy: $V_{13}=m_{13}g\left[\ell_{u}\left(1-\cos\phi_{1}\right)+{\overset{¯}{r}}_{13}\left(1-\cos\phi_{2}\right)\right]$

The total kinetic energy of this system is now given with

(7) $$T=T_{1}+T_{2}+2\left(T_{3,1}+T_{3,2}+T_{3,3}\right)+T_{4}+2T_{5}+T_{6}+T_{7}+\left(T_{8,1}+T_{8,2}+T_{8,3}+T_{8,4}\right)+T_{9}+\sum T_{10,i}+\sum T_{11,i}+T_{12}+T_{13},$$

and the total potential energy is given with

(8) $$V=V_{1}+V_{2}+2\left(V_{3,1}+V_{3,2}+V_{3,3}\right)+V_{4}+2V_{5}+V_{6}+V_{7}+\left(V_{8,1}+V_{8,2}+V_{8,3}+V_{8,4}\right)+V_{9}+\sum V_{10,i}+\sum V_{11,i}+V_{12}+V_{13}\text{.}$$

By combining the potential and kinetic energies, the total energy as a function of $\phi_{1},\phi_{2},{\overset{˙}{\phi }}_{1}$, and ${\overset{˙}{\phi }}_{2}$ is

(9) E=T+V

However, to determine the EOM, Lagrange’s equation will be implemented, which requires the Lagrangian. The Lagrangian of the system is defined as

(10) L=T-V

Lagrange’s equations is then applied to the system to for the equation of motion for the upper and lower link as

(11) $$\begin{array}{c} \frac{d}{\mathrm{dt}}\left(\frac{\partial L}{\partial \overset{˙}{\phi_{1}}}\right)-\frac{\partial L}{\partial \phi_{1}}+M_{v}^{\left(i\right)}+M_{q}^{\left(i\right)}+M_{c}^{\left(i\right)}=0,\\ \frac{d}{\mathrm{dt}}\left(\frac{\partial L}{\partial \overset{˙}{\phi_{1}}}\right)-\frac{\partial L}{\partial \phi_{1}}+M_{v}^{\left(i\right)}+M_{q}^{\left(i\right)}+M_{c}^{\left(i\right)}=0, \end{array}$$

where $M_{v},M_{v}$, and $M_{v}$ are the damping moments from viscous, quadratic, and Coulomb damping.

### A.1 Bearing Kinematics

Let $d_{I}$ be the diameter of the inner race of the ball bearing, $d_{B}$ the diameter of the balls, $\omega_{D}$ the angular velocity of axis i^-j^ which is attached to ${\overset{¯}{\mathrm{OC}}}_{0},\omega_{A}$ the angular velocity of the inner race of the bearing, and $\omega_{c}$ is the angular velocity of the ball bearing. Consider the velocity of point *P* on the inner race

(12) v→P=v→0+v→P/O=v→0+ωAdI2i^.

If we now consider the velocity of point P′ on the ball bearing which is in contact with point *P* at the shown instant, we obtain

(13) $${\overset{\to }{v}}_{P\prime }={\overset{\to }{v}}_{C_{0}}+{\overset{\to }{v}}_{P\prime /C_{O}}\text{.}$$

But v→C0=v→0+v→C0/O=v→0+12(dI+dB)ωDi^, so we can write

(14) v→P′=v→0+12(dI+dB)ωD+ωcdBi^.

Using the no-slip condition implies that $v_{P}=v_{P\prime }$, which gives

(15) $$\omega_{A}d_{I}=(d_{I}+d_{B})\omega_{D}+d_{B}\omega_{C}\text{.}$$

If we now consider the velocity of point C′ on the ball bearing, we can write

(16) v→C′=v→C0+vC′/C0=v→0+12(dI+dB)ωD-ωcdBi^.

Point C′ is in contact with point *C* on the outer race whose velocity is given by

(17) v→C=v→O+v→C/O=v→0+(dI+2dB)2ωBi^

Using the no-slip condition between points *C* and C′ leads to $v_{C}=v_{C\prime }$, which gives

(18) $$(d_{I}+d_{B})\omega_{D}-d_{B}\omega_{C}=(d_{I}+2d_{B})\omega_{B}\text{.}$$

Adding Eqs. (15), (18) gives

(19) $$\omega_{D}=\frac{d_{I}}{2(d_{I}+d_{B})}\omega_{A}+\frac{d_{I}+2d_{B}}{2(d_{I}+d_{B})}\omega_{B}\text{.}$$

Substituting the expression for $\omega_{D}$ from Eq. (19) into Eq. (15) gives

(20) $$\omega_{C}=\frac{d_{I}}{2d_{B}}\omega_{A}-\frac{\left(d_{I}+2d_{B}\right)}{2d_{B}}\omega_{B}\text{.}$$

The angular velocities of the inner race $\omega_{A}$ and the outer race $\omega_{B}$ depend on the double pendulum design. Fig. 20 shows two alternatives: Fig. 20a is the pendulum design we presented in this manuscript with the inner race fixed to the upper link, while the bottom link is attached to the outer race of the pendulum. For the design used in this paper we have $\omega_{A}={\overset{˙}{\phi }}_{1}$ and $\omega_{B}={\overset{˙}{\phi }}_{2}$. Substituting these expressions into Eq. (20), (19) results in

(21) $$\begin{array}{cc} \omega_{C} & =\frac{d_{I}}{2d_{B}}{\overset{˙}{\phi }}_{1}-\frac{\left(d_{I}+2d_{B}\right)}{2d_{B}}{\overset{˙}{\phi }}_{2},\\ \omega_{D} & =\frac{d_{I}}{2\left(d_{I}+d_{B}\right)}{\overset{˙}{\phi }}_{1}+\frac{d_{I}+2d_{B}}{2\left(d_{I}+d_{B}\right)}{\overset{˙}{\phi }}_{2}\text{.} \end{array}$$

In contrast, Fig. 20b shows an alternative pendulum design where the axle holding the bottom link is attached to the inner race giving $\omega_{A}={\overset{˙}{\phi }}_{2}$ and $\omega_{B}={\overset{˙}{\phi }}_{1}$. Table 5 shows the resulting $\omega_{D}$ and $\omega_{C}$ expressions for each configuration. Additionally, the resulting expressions for the angular velocities are provided in Table 5, where the left column is for the configuration provided in this manuscript and the right is the alternative design.

## Appendix B Data collection and analysis

### B.1 Encoder data collection and calibration

In this section, the encoder data collection and calibration is discussed. To begin the data collection process, the encoder was wired. To do this, the output from the encoder was connect to an analog input pin from on the DAQ and the encoder was power with 24 V with the power supply. The output from the encoder ranges from 0–10 V with approximately 0.65 V per full revolution. This encoder allows for the pendulum to rotate multiple times without exceeding the rotational bounds. The recording rate was set to 500 Hz to sufficiently capture the dynamics of the double pendulum.

The only step needed to analyze the raw voltage data is to calibrate the encoder between angle and voltage. To do this, seven data points were taken at intervals of full rotations from an original downward configuration considered zero position at approximately the 5 V location. At each downward configuration, it is assumed that the angle has no error and thus include the angles ϕ=[-1080°,-720°,-360°,0°,360°,720°,1080°]. The corresponding voltage at each downward configuration was then recorded for 3 s at a rate of 500 Hz to allow for the calculation of the mean and standard deviation at each. The resulting calibration points are shown in Fig. 21, which shows a highly linear relationship between voltage and angle. Additionally, a zoomed in point at the positive 360° location shows that the standard deviation of the voltage falls within the 2.5 mV specified uncertainty of the encoder.

### B.2 Pendulum error analysis for video data

For simplicity, we will only show the error analysis for the single pendulum as the general rule will apply to both single and double pendulums. For our analysis, we will compare two ways of determining ϕ. The first (see left of Fig. 22) directly measures ϕ from a reference vector $v_{\mathrm{ref}}$, and the second (see right of Fig. 22) uses the change in angle between consecutive frames Δϕ with ϕ being calculated using the summation of all previous Δϕ over a total of *T* frames as $\phi =\sum_{i}^{T}\Delta \phi_{i}$. While both methods will work for determining ϕ if there is no noise in the system, it was found that the first method (left side of Fig. 22) avoids a compounding error, which is prevalent even with small amounts of uncertainty or noise.

### B.3 Pendulum ϕ measurements using video data

This section describes the reasoning for the method used for calculating the upper link’s angle to vertical $\phi_{1}$ and the lower links angle to vertical $\phi_{2}$ of a double pendulum as shown on the right side of Fig. 23. To begin, a node assignment is placed on each marker on the pendulum for reference to which rigid body the node belongs to (see left side of Fig. 23) with the upper link having $[n_{0},n_{1},n_{2}]\in L_{1}$ and the lower link having $[n_{3},n_{4},n_{5},n_{6},n_{7},n_{8}]\in L_{2}$. Using the rigid body node assignment, position vectors between nodes for the upper and lower links are calculated as

(22) $$\begin{array}{cc} {\overset{\to }{p}}_{j/i}(t) & =[x_{j}(t)-x_{i}(t),\;y_{j}(t)-y_{i}(t)]\\ & =[x_{j/i}(t),\;y_{j/i}(t)] \end{array}$$

where only the direction is considered if the source node $n_{i}$ and sink node $n_{j}$ are such that j>i and nodes $n_{i}$ and $n_{j}$ are part of the same link. This is done to avoid repeated vectors that are opposite in direction. Using all the possible vector combinations in each link, the angle between the original position vector ${\overset{\to }{p}}_{j/i}(0)$ and the configuration at a later time ${\overset{\to }{p}}_{j/i}(t)$ is used to calculate the angle between the two as

(23) $$\phi_{L}^{\left(i,j\right)}(t)=\tan^{-1}\left(\frac{y_{j/i}(t)}{x_{j/i}(t)}\right)-\tan^{-1}\left(\frac{y_{j/i}(0)}{x_{j/i}(0)}\right),$$

where tan is arctan2 to allow for the correct angle sign for all quadrants. A toy example of this process is shown in Fig. 24 with the vector from node $n_{3}$ to node $n_{4}$ at time *t* and t=0 with a down configuration. This process is applied for all configuration and the mean angle $\mu_{\phi }(t)$ and standard deviation $\sigma_{\phi }(t)$ are calculated calculated as

(24) $$\begin{array}{c} \mu_{\phi }(t)=\frac{1}{N}\underset{\left(i,j\right)}{\sum }\phi_{L}^{\left(i,j\right)}(t),\\ \sigma_{\phi }(t)=\sqrt{\frac{1}{N}\underset{\left(i,j\right)}{\sum }{\left[\mu_{\phi }(t)-\phi_{L}^{\left(i,j\right)}(t)\right]}^{2}}\text{.} \end{array}$$
